# Supplementary material for: Down Regulation and Loss of Auxin Response Factor 4 Function Using CRISPR/Cas9 Alters Plant Growth, Stomatal Function and Improves Tomato Tolerance to Salinity and Osmotic Stress
Source: Genes (Basel). 2020 Mar 3;11(3):272. doi: 10.3390/genes11030272 (PMC7140898; doi:10.3390/genes11030272)
Supplement: Supplementary file 1 [file genes-11-00272-s001.pdf]

Supplementary material

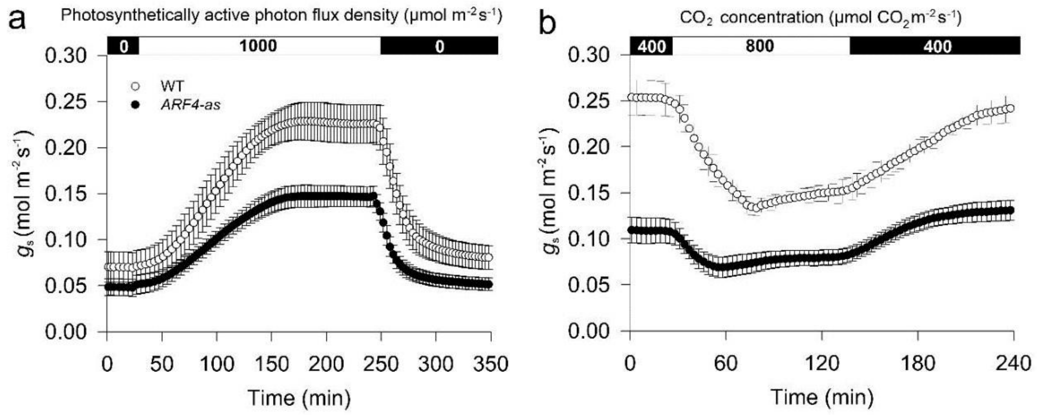

Figure S1. Stomatal responses to irradiance and CO_2 levels in Micro-Tom (WT) and *ARF4*-as transgenic line. (a) Stomatal conductance (g_s) response in tomato plants cv. Micro-Tom (WT) and isogenic *ARF4* antisense transgenic line (*ARF4*-as) from dark-adapted (0 μmol m^{-2} s^{-1}) leaves exposed to light (1000 μmol m^{-2} s^{-1}) and on subsequent transfer back to darkness (0 μmol m^{-2} s^{-1}) in 350 minutes interval (n=5). (b) Stomatal conductance in response to CO_2 elevation and subsequent decrease to ambient CO_2 (400-800-400 μmol CO_2 m^{-2} s^{-1}) in 240 minute interval (n=4). Measurements were performed using a LI-6400; LI-COR gas exchange chamber in plants aged 40 days after germination. Data presented are mean ± SE obtained using the 5th leaf totally expanded.

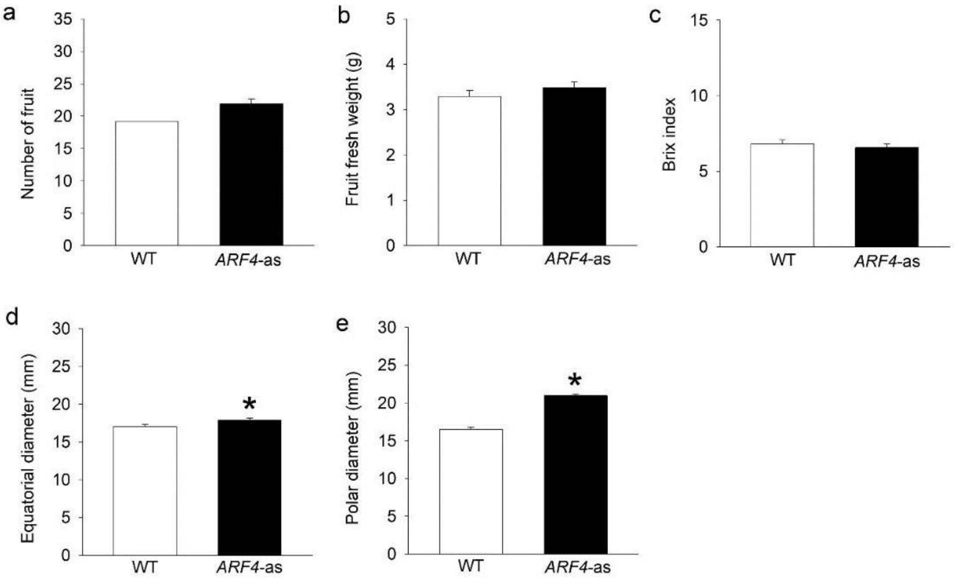

Figure S2. Productive parameters in tomato Micro-Tom (WT) and *ARF4*-as transgenic line. (a) Number of fruit, (b) Fruit fresh weight, (c) brix index, (d) equatorial diameter and (e) polar diameter of the fruit. Values are means ± s.e.m (n=8 plants).Asterisks indicate values that were determined by Student's t test to be significantly different ($P < 0.05$) from Micro-tom (WT).

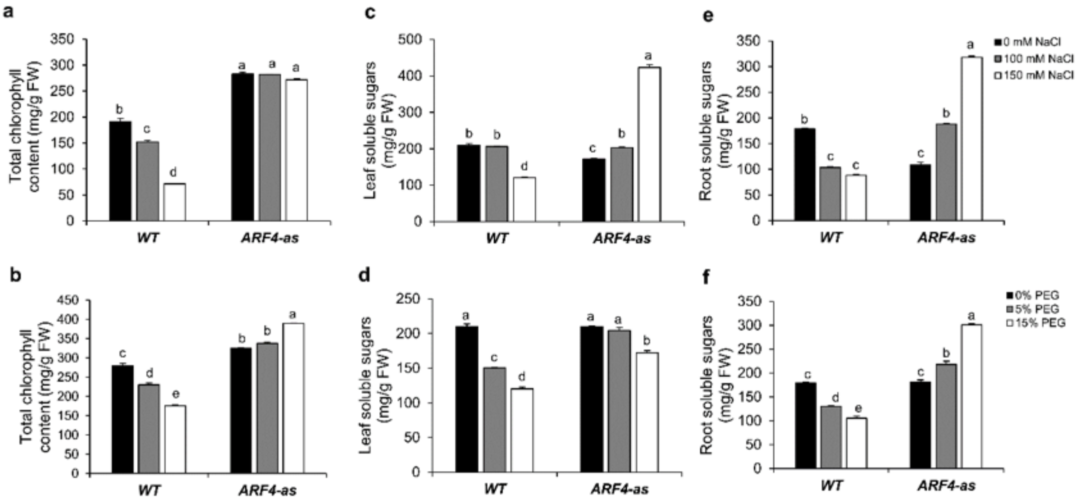

Figure S3. Photosynthesis and sugar accumulation in WT and *ARF4*-as plants exposed to different concentrations of NaCl or PEG. (a) and (b) total chlorophyll content in salt and osmotic stress conditions respectively, (c) and (d) leaf soluble sugars in salt and osmotic stress conditions respectively, (e) and (f) root soluble sugars content in salt and osmotic stress conditions respectively. Salt and osmotic stresses were performed on three weeks tomato plants for two weeks by adding 100mM of NaCl or 150 mM of NaCl for salt stress or 5% or 15% of PEG 20 000 for osmotic stress. Values are mean ± SD of three biological replicates. Bars with different letters indicate the statistical significance (p<0,05) according to Student Newman-Keuls test.

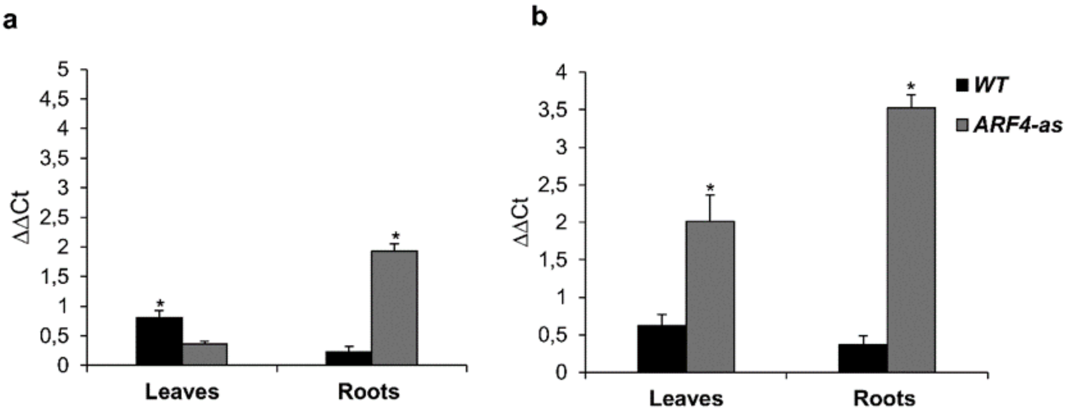

Figure S4. Expression of sucrose transporter *SlSUT1* in WT and *ARF4*-as plants exposed to salt or osmotic stresses. (a) gene expression in leaves and roots exposed to 150mM of NaCl, (b) gene expression in leaves and roots leaves exposed to 15% PEG. ΔΔCt refers to fold differences in gene expression relative to untreated plants. Values are mean ± SD of three biological replicates. Stars (*) indicate the statistical significance (p<0,05) according to Student's t-test.

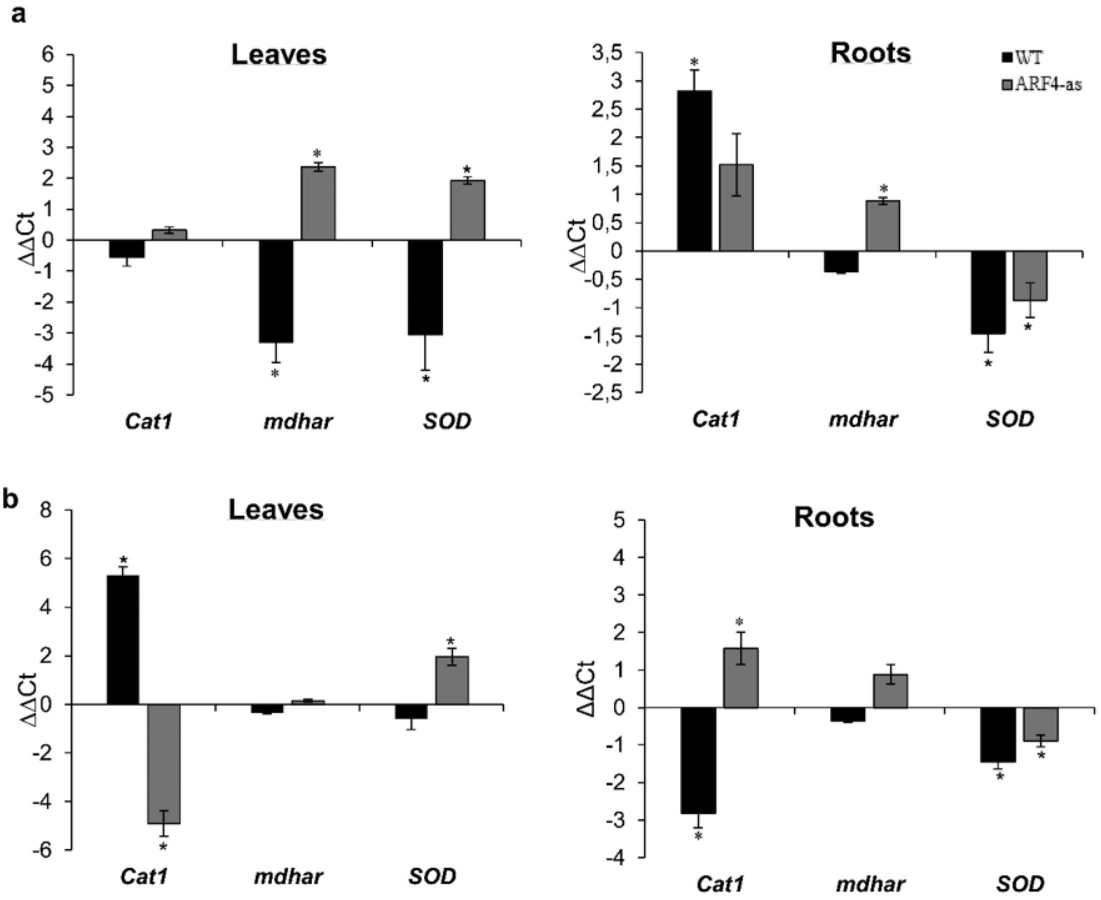

Figure S5. Expression of *Cat1*, *mdhar* and *SOD* in WT and *ARF4*-as plants exposed to salt or osmotic stresses**.** (a) gene expression in leaves and roots exposed to 150mM of NaCl, (b) gene expression in leaves and roots leaves exposed to 15% PEG. ΔΔCt refers to fold differences in gene expression relative to untreated plants. Values are mean ± SD of three biological replicates. Stars (*) indicate the statistical significance (p<0,05) using Student's t-test.

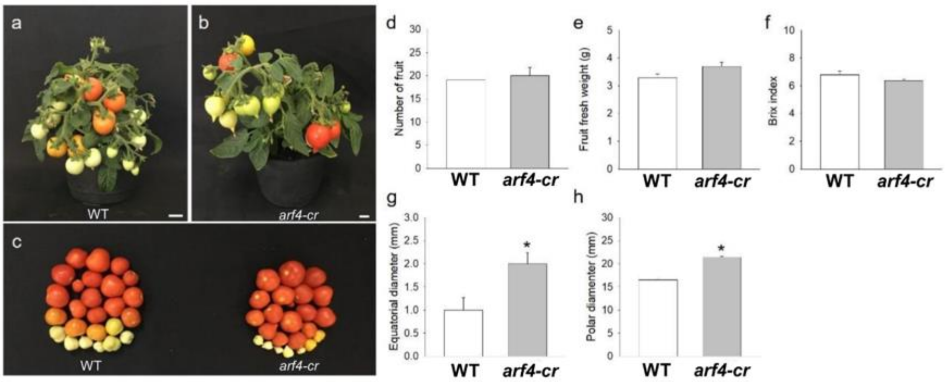

Figure S6. Productive parameters in tomato Micro-Tom (WT) and *arf4-cr* transgenic line**.** (a) and (b) representative tomato plants in reproductive stage, (c) fruit yield, (d) Number of fruit, (e) Fruit fresh weight, (f) percentage of soluble solids, (g) Equatorial diameter of the fruit and (h) Polar diameter of the fruit. Values are means ± s.e.m (n=8). The brix index was measured in 10 fruits per repetition. Asterisks indicate values that were determined by Student's t test to be significantly different ($P < 0.05$) from wild-type (WT).

Table S1. Gene ID and quantitative RT-PCR primers.

Gene	Solyc ID	Forward primer sequence	Reverse primer sequence
Sl-ARF4	Solyc11g069190	CATTATTGTTGGTGACTTTGTG	GACCTTTGGAAACCTATTGG
Cat1	Solyc12g094620.1.1	ACCCGATTCCTTCTTGTGTC	TGATGTATCTGTCTTGCCTGTC
mdhar	Solyc08g081530.2.1	CGGATTTCAAGGGTTTCGGTTC	CTCCTCCAACTACCACATACTCTC
SOD	Solyc01g067740.2.1	ATCAGCACTCATATTGGACTTCTC	TGCCACTAACACCTTCACTG
SlSUT1	Solyc11g017010.1.1	CTGGGATGATTTGTTTGGAGGA	GGTTTAGCATCAGCAGGTGG
SlNCED1	Solyc07g056570.1.1	GATTGTTTCTGTTTCCACCTCTG	ACACTCTTTAGCCCTTCATCAC
SlNCED2	Solyc08g016720.1.1	GGCATAACCTTCCCGTTACAG	AAAGATGATGACCGGCAACC
SlCYP707A1	Solyc04g078900.2.1	CCGAAACCCAATACATTTATGCC	ATCCTACCACTTCCCACCTG
SlCYP707A2	Solyc08g005610.2.1	CAACAAAGCAATGAAAGCGAGG	CAGTGAGTCCTTCTTTATCTCCC
SlCYP707A3	Solyc08g075320.2.1	CCTAGTGTCCTACAAGCTGTC	GCAGCTCTAAGTGTCTCTTGG
Sl-Actin	Solyc03g078400	TGTCCCTATCTACGAGGGTTATGC	AGTTAAATCACGACCAGCAAGAT

Table S2. Characterization of photosynthetic parameters in tomato cv. Micro-Tom (WT) and isogenic *ARF4* antisense transgenic line (*ARF4*-as) Vcmax: maximum Rubisco carboxylation rate; Jmax: maximum electron transport rate; TPU: triose phosphate utilization. Mean values (n=5) ± s.e.m. Significant differences by t-test at 0.01.

	**WT**	***ARF4*-as**
**V_{cmax} (μmol m^{-2} s^{-1})**	80.37 ± 2.34	70.43* ± 3.01
**J_{max} (μmol m^{-2} s^{-1})**	156.40 ± 7.74	140.70* ± 6.30
**TPU (μmol m^{-2} s^{-1})**	11.84 ± 0.57	10.82 ± 0.53
